# Supplementary material for: Are carbon emissions trading and green financial instruments synergistic? -Comprehensive quantitative research based on content analysis
Source: PLoS One. 2024 Mar 7;19(3):e0298601. doi: 10.1371/journal.pone.0298601 (PMC10919601; doi:10.1371/journal.pone.0298601)
Supplement: S1 Data — (ZIP) [file pone.0298601.s001.zip › Data/地级市Z_F工作报告.zip!/║╙─╧/Σ≡║╙╩╨/└┤╫╘╣1⁄2╓┌║┼í╛┬φ┐╦╩2╛▌═°í┐_2007.docx]

各位代表:

现在,我代表市人民政府,向大会作政府工作报告,请予审议,并请各位政协委员提出意见。

折叠编辑本段过去六年回顾

--经济持续快速发展,综合实力跨上新台阶。2006年,全市生产总值377亿元,比2000年翻了一番;人均生产总值14869元,高于全省平均水平1590元;地方财政一般预算收入16.1亿元,是2000年的2.8倍;社会消费品零售总额109.5亿元,是2000年的2倍;城镇居民人均可支配收入9034元,农民人均纯收入3883元,分别比2000年增加4401元和1580元;金融机构各项存款余额231.4亿元,比2000年增加112亿元。

--经济结构不断优化,产业优势更加凸显。三次产业结构调整为16.8:64.5:18.7,由二、一、三演进为二、三、一格局。工业增加值占全市生产总值的61.4%,比2000年提高11.9个百分点。重点工业300亿振兴计划、500亿上台阶工程顺利完成。以双汇集团为龙头的食品产业优势日益明显,我市成为首家中国食品名城、全国食品安全信用体系和保证体系建设双试点市、全省食品工业基地市、全省无公害食品基地示范市。国家肉类及制品质量监督检验中心建在我市。造纸工业快速膨胀,盐化工业发展迅猛,纺织、机械制造等产业不断壮大。农业结构进一步优化,优质粮食种植面积占70%以上,畜牧业增加值占农业增加值的42%,农业产业化重点龙头企业数量、无公害农产品产地认定和产品认证数量均居全省前列,我市荣获全国农业产业化先进集体称号。服务业发展态势良好,区域性商贸物流中心建设取得重要进展。

--改革开放成果丰硕,发展后劲显著增强。企业改革成效明显。双汇国有产权成功转让,双汇集团国际化发展迈出了关键性一步;银鸽股权分置改革顺利完成;澳的利等一批企业靠大联大,实现了快速发展;一些改制企业的遗留问题得到较好解决。非公有制经济快速发展,完成增加值占全市生产总值的54%。事业单位改革逐步深化,农村综合改革深入推进,分配制度改革有序展开,财税、金融、投资、供销、粮食流通等方面的改革取得重大进展。行政机构改革和行政审批制度改革不断深化,市行政服务中心建成投用。整顿和规范市场秩序工作富有成效。对外开放迈出新步伐。连续成功举办了四届中原食品节,组织了长三角、珠三角和上海食品名城推介等重大招商活动。累计审批外资企业122家,实际利用外资3亿美元,现有20多个国家和地区的客商在我市投资发展,6家世界500强和一批知名企业落户漯河;年外贸出口近亿美元,鲜冻猪肉出口占全省的90%以上、占全国的四分之一。双汇三期工程等40多个重大工业项目建成投产,新天地商业步行街等五大重点市场建成投用。全社会固定资产投资累计完成473亿元,其中工业投资170亿元,有力地拉动了全市经济快速增长。

--城镇化进程明显加快,新农村建设扎实推进。成功实施了新的行政区划调整,掀开了漯河发展史上崭新的一页。城市规划、建设和管理迈上新台阶,编制了城市总体规划及覆盖全市60%的控制性详规,市区建成区面积由33.5平方公里发展到48.6平方公里,常住人口由33万人增加到46万人,全市城镇化率达到33.3%,六年提升12个百分点。走出了经营城市新路子,搭建了新的投融资平台。新区建设和旧城改造成效明显,新建改造市区道路62条,南环、北环、东环建成通车,整治背街小巷近百条,4个入市口改造全面完成,供水供热、燃气输配、垃圾处理、污水处理等一批公用设施建成投用。"双创"工作深入开展,市区沿街综合整治成效显著,绿化、美化、亮化水平得到提升,市容市貌明显改观,我市成为国家园林城市、全国绿化模范城市、全省造林绿化最佳城市,荣获中国人居环境范例奖。漯驻、漯周、漯平高速公路建成通车,实施了107国道漯河段和漯舞、漯阜铁路漯河段改造,开通了漯河至上海的始发列车。完成了城乡电网改造工程,漯河500千伏变电站等电力设施建成投运。通信基础设施建设不断加强。临颍、舞阳两县城区规模扩大,小城镇建设特色明显,4个镇被确定为国家级重点镇。新农村建设扎实推进,"两县十镇百村示范带动工程"顺利实施。农业税全免、粮食直补、良种补贴、农机购置补贴等支农惠农政策全面落实,政策性减轻农民负担4.8亿元。累计转移农村劳动力35.1万人。新建改建农村公路2431公里,在全省率先实现"村村通"目标。发展户用沼气池8.5万座。解决了14万人饮水安全问题。新建改造"农家店"392家。累计投入5800万元,新建改造"温暖小区"605个,在全国率先解决农村五保户住房难问题。农村五保户集中供养率达到52%,高于省定目标32个百分点。我市被确定为国家农村信息化综合信息服务试点市。农田水利建设、农业综合开发和扶贫开发、农作物秸秆禁烧及综合利用、人工影响天气等工作实现新的突破。

--社会事业全面进步,和谐漯河建设成效明显。建立了中科大(漯河)科技园,与江南大学建立了全面合作关系,"九漯合作"取得阶段性成果。建成了国家级高新技术创业服务中心、13家省级以上企业技术中心,新创省级以上名优产品46个、中国驰名商标和河南省著名商标21个。"全国品牌万里行"把我市作为活动站点。人才工作不断加强,各类人才作用得到发挥。教育事业快速发展,我市成为全国教改实验区,6所高中被命名为全省示范性高中,漯河卫校升格为医学高等专科学校,漯河职院被评为全国十大特色高职院校;累计发放农村贫困家庭学生"两免一补"资金4100万元,在全省率先实现农村中小学现代远程教育"校校通",完成了372个农村中小学D级危房改造项目。新建改造了一批市县医院和40所乡镇卫生院,城镇医保覆盖面不断扩大,新型农村合作医疗制度全面推行,公共卫生服务体系更加完善。我市成为全国首家无白内障障碍示范市。文化旅游产业发展势头良好,南街村景区被命名为4A级景区,新增国家重点文物保护单位6处、省级文物保护单位11处。就业再就业和社会保障工作力度加大,累计新增城镇就业人员16.7万人,下岗失业人员再就业8.5万人,农村最低生活保障制度全面实行,城镇低保对象实现应保尽保。住房公积金个人贷款使用率、收益率均居全省首位。廉租住房建设走在全省前列。"双清欠"任务圆满完成。市体育馆、图书馆、老干部活动中心、市一院新病房大楼、人防应急中心等建成投用。广电、审计、统计、史志档案、民族宗教、外事侨务等事业全面发展,人防、老龄、群众体育、关爱女孩等工作走在全国前列。人民生活水平大幅提高。城乡居民人均储蓄6623元,比2000年增加2832元;城乡居民人均居住面积均高于全省平均水平;文化、教育、娱乐消费支出比2000年提高3.7个百分点;城乡居民的衣食住行都发生了可喜变化。

市人大及其常委会的各项决议、决定得到全面落实,人大代表议案、建议和政协委员提案办结率、满意率均达到99%以上。第四、第五届村委会换届选举顺利完成。我市成为全国普法依法治理先进城市。群众性精神文明创建活动深入开展,全民素质和文明程度不断提高。双拥工作和国防教育深入开展,荣获全省双拥模范城,驻漯解放军、武警和民兵预备役在抗洪抢险、维护社会稳定、支持地方经济建设中作出了重要贡献。抗击非典保持"双零"记录,"7?17"特大洪灾无一人伤亡,依法稳妥处理了银鹰典当行非法集资案,集中打击了非法传销活动,最大限度地保护了广大群众的生命财产安全和切身利益。计划生育、环境保护和土地管理等国策全面落实,我市荣获全省人口与计划生育工作先进市、全省环保先进单位。党风廉政建设得到加强,政风行风不断好转。社会大局持续稳定,我市成为全国信访工作先进集体、全省安全生产优秀市、全省社会治安综合治理先进市。

各位代表!过去六年,全市人民用辛劳和智慧、激情与拼搏,谱写了漯河发展史上的精彩篇章。这六年,是我市综合实力显著增强的时期,是产业特色日益凸显的时期,是城乡面貌发生重大变化的时期,是人民群众得到实惠较多的时期。今天的漯河,正在成为一个充满生机、充满活力、充满希望的城市。

六年来的成绩来之不易,这是省委、省政府和市委正确领导的结果,是市人大、市政协监督支持的结果,是全市人民团结奋斗的结果。在此,我代表市政府,向全市工人、农民、知识分子和各级干部,向驻漯解放军指战员、武警官兵和公安民警,向所有关心支持漯河发展的各界人士,表示衷心的感谢!

在肯定成绩的同时,我们也清醒地看到,全市经济社会发展中还存在一些问题:经济实力还不够强,经济结构还不尽合理,第三产业比重较低,高新技术产业发展缓慢;固定资产投资总量相对较小,重大建设项目还不够多;县区经济发展还不够快;城市管理水平有待提高;水污染防治工作需要继续加强;经济发展环境有待进一步优化;一些影响稳定的因素仍然存在。我们一定采取更加有力的措施,切实解决这些问题,决不辜负人民的期望。

折叠编辑本段今后奋斗目标

今后五年,是扎实推进富强漯河、和谐漯河建设的关键时期。我们面临着难得的发展机遇,世界经济正处于新一轮增长期,我国经济继续保持平稳较快增长,国际国内产业转移加快,国家对中部地区和新农村建设支持力度加大,中原城市群建设快速推进,区域间合作更加紧密。我市经过20年发展,奠定了良好的基础;市五次党代会描绘了漯河未来五年的宏伟蓝图,发展方向更加明确,发展重点更加突出,全市上下加快发展的愿望更加强烈,具备了跨越发展的有利条件。同时,我们也面临着更大的压力和挑战,周边地区特别是中原城市群各市你追我赶,发展形势咄咄逼人。我们必须进一步增强加快发展的使命感、责任感和紧迫感,顺天时、应地利、聚人和,抢抓机遇,乘势而上,立足新起点,再创新辉煌。

今后五年政府工作的总体要求是:以邓小平理论和"三个代表"重要思想为指导,全面落实科学发展观,紧紧围绕市五次党代会确定的打造"两个名城"、推进"三大建设"、加快实现"两个率先"的总体奋斗目标,着力调整经济结构和转变增长方式,着力加强资源节约和环境保护,着力推进改革开放和自主创新,着力促进社会发展和解决民生问题,加快推进工业化、城镇化和农业现代化,加快发展各项社会事业,加快建设富强漯河、和谐漯河,努力实现又好又快发展,使漯河更加充满生机、更加充满活力、更加充满希望。按照这一要求,今后五年的奋斗目标是:

--经济发展走在全省前列。经济质量大幅提高,经济结构优化升级,经济布局更趋合理,经济实力显著增强。生产总值、规模以上工业增加值、地方财政一般预算收入、城镇固定资产投资年均增速居全省上游。2011年,力争全市经济总量翻一番。

--新农村建设走在全省前列。农村经济全面发展,现代农业加快推进,农民收入大幅增加,生产生活条件显著改善,文化生活丰富多彩,基层管理更加民主。

--城乡一体化走在全省前列。力争成为全省城乡一体化试点市,中心城区、县城和重点小城镇的带动作用明显增强,城乡互动,共同发展,城乡基础设施更加完善,城乡居民共享城市文明,在全省率先实现城乡一体化。

--和谐社会建设走在全省前列。各项社会事业繁荣进步,社会保障体系日益完善,居民生活质量明显提高,民主法制更加健全,公平正义得到弘扬,幸福指数不断提升。把漯河建成全省最干净的城市、全省最平安的城市,力争成为全国科技进步先进市、全国文明城市、国家卫生城市、全国环保模范城市、中西部地区最适宜创业发展和居住的城市。

实现上述目标,要突出抓好六项重点:

(一)致力建设工业大市和工业强市,努力打造最具竞争力的中国食品名城。工业兴、则漯河兴,工业强、则漯河强,漯河大发展,关键在工业。我们必须坚持工业强市不动摇,更加突出工业的核心地位,把快速扩张工业总量、转变增长方式作为经济工作的重中之重,进一步营造关心工业、支持工业、大办工业、办大工业的浓厚氛围。立足产业特色,做大做强食品工业,快速发展造纸工业,着力培育盐化工业,加快建设最具竞争力的中国食品名城、中西部地区重要的造纸基地和全省重要的盐化工基地。2011年,全市食品工业销售收入突破1100亿元,肉制品年加工生产能力200万吨,粮食加工转化能力500万吨,造纸生产能力160万吨,盐化工产品产量700万吨。坚持抓大育大,培植骨干群体。2011年,全市要培育出年销售收入超500亿元的企业1家、超100亿元的企业3家、超50亿元的企业5家和一批超10亿元企业。大力实施中小企业成长工程,引导中小企业向专、精、特、新方向发展,重点扶持一批高成长性中小企业迅速壮大。加快培育一批上市备选企业,力争早日上市,增强企业融资能力。鼓励和支持企业引进高新技术,积极推广先进适用技术,不断提高技术创新能力。深入实施名牌战略,培育更多的知名品牌。2011年,规模以上工业企业中,60%的企业与高等院校、科研机构建立产学研协作关系;全市省级以上名牌产品达到50个以上、中国驰名商标和河南省著名商标50个以上。积极培育具有潜力的新兴产业,大力发展产业集群,带动全市工业实现新的跨越。今后五年工业累计完成投资达到350亿元,2011年全市工业销售收入达到1800亿元。

(二)奋力开创改革开放新局面,全面推动开放的漯河更开放。漯河过去的发展,得益于改革开放;今后的发展,更需要改革开放。我们要重振开放雄风,让解放的思想更解放、开放的漯河更开放,为新一轮大发展增添强大动力和活力。加大改革攻坚力度,不断把经济、社会、文化等各个领域的改革向纵深推进、向广度拓展。完善更加优惠的对外开放政策,营造更加优良的对外开放环境,采取更加有效的对外开放举措,努力实现招商引资和出口创汇新突破。今后五年,全市累计实际利用外资6亿美元以上,出口总额年均增长20%以上。积极开展区域合作,主动承接产业转移,注重加强与长三角、珠三角和中原城市群之间的协作,努力实现优势互补、合作共赢。

(三)大力发展现代农业,扎实推进社会主义新农村建设。按照"生产发展、生活宽裕、乡风文明、村容整洁、管理民主"的要求,科学规划,分类指导,典型引路,梯次推进,确保新农村建设扎实有效。以发展现代农业为重点,用现代物质条件装备农业,用现代科学技术改造农业,提高农业机械化程度和产业化经营水平。不断加大对农业农村的投入力度,加快农村基础设施建设和社会事业发展,着力改善生产生活条件。发展壮大县区经济,增强县区综合实力,拓宽农民增收渠道,切实增加农民收入。2011年,全市优质粮食种植面积达到88%以上,畜牧业增加值占农业增加值的50%以上;农民人均纯收入达到5400元以上;培育出10个以上强乡镇和150个以上强村;70%以上的村通班车;35%以上的村用上自来水,60%的农户用上沼气;农村有线电视覆盖率达到80%;"万村千乡市场工程"覆盖率达到100%。

(四)着力发展服务业,加快建设区域性商贸物流中心。大力发展面向生产、生活的服务业。充分发挥我市的区位交通优势、产业特色优势和传统商业优势,重点发展商贸业和现代物流业,繁荣各类商品市场、要素市场特别是大型专业批发市场,规划建设大型物流基础设施,培育发展连接郑州、带动豫南的综合物流园区。加快发展会展经济,逐步把中原食品节办成中国食品节。重视发展文化旅游产业,办好许慎文化国际研讨会,使漯河逐步成为国际性许慎文化研究中心、许学文献资料中心。有序发展房地产业,增加普通商品房和经济适用房供应。积极发展信息咨询、金融保险、社区服务等现代服务业,逐步提高第三产业占生产总值的比重。

(五)强力推进城乡一体化,加快建设富有魅力的生态宜居名城。全面加强城市建设和管理,努力实现城市规划科学化、城市建设集约化、城市管理现代化。强化中心城区建设,理顺城区管理体制,实行规划一张图、审批一支笔、建设一盘棋、管理一个法,塑造雅气、秀气、灵气、大气的城市品位,彰显河流、森林、花园、精品建筑交相辉映的城市风貌。高标准完成沙澧河沿岸市区段开发建设,把沙澧河沿岸建成风景长廊、生态长廊、文化长廊和休闲长廊,使沿河景观带成为展示漯河形象的重要标志,使城市价值、特色优势充分显现。全面推进基础设施向农村延伸、公共服务向农村拓展、现代文明向农村辐射、社会保障向农村覆盖,加快构建新型城乡关系,促进城乡一体化水平快速提高。2011年,全市城镇化率达到43%,市区建成区面积达到60平方公里以上、常住人口60万人以上。

(六)全力解决民生问题,加快构建和谐漯河。坚持以人为本,更加关注民生。优先发展教育事业,巩固义务教育成果,普及高中阶段教育,加快发展职业教育,不断提升高等教育,让更多的人享有更好的教育。完善城乡卫生服务和医疗保健体系,健全突发公共卫生事件监测和预警系统。把扩大就业放在经济社会发展的突出位置,促进城乡劳动者充分就业。高度重视解决农民工问题,使农民工与城市职工享有平等的待遇和权益。加快建立覆盖城乡的社会保障体系,切实解决困难群体住房、就业、就医、子女上学等难题。进一步加强食品药品监管,保障人民群众饮食和用药安全。做好社会治安综合治理和安全生产工作。正确处理经济增长与人口、资源、环境的关系,积极发展循环经济,建设资源节约型和环境友好型社会,努力实现可持续发展。
